# Scientific sinkhole: estimating the cost of peer review based on survey data with snowball sampling

**DOI:** 10.1186/s41073-023-00128-2

**Published:** 2023-04-24

**Authors:** Allana G. LeBlanc, Joel D. Barnes, Travis J. Saunders, Mark S. Tremblay, Jean-Philippe Chaput

**Affiliations:** 1 Healthy Active Living and Obesity Research Group, CHEO Research Institute, Ottawa, ON Canada; 2 Ottawa, ON Canada; 3 Department of Applied Human Sciences, University of Prince Edward Island, Charlottetown, PE Canada

**Keywords:** Cost, Peer-review, Publishing, Manuscript, Article, Paper

## Abstract

**Background:**

There are a variety of costs associated with publication of scientific findings. The purpose of this work was to estimate the cost of peer review in scientific publishing per reviewer, per year and for the entire scientific community.

**Methods:**

Internet-based self-report, cross-sectional survey, live between June 28, 2021 and August 2, 2021 was used. Participants were recruited via snowball sampling. No restrictions were placed on geographic location or field of study. Respondents who were asked to act as a peer-reviewer for at least one manuscript submitted to a scientific journal in 2020 were eligible. The primary outcome measure was the cost of peer review per person, per year (calculated as wage-cost x number of initial reviews and number of re-reviews per year). The secondary outcome was the cost of peer review globally (calculated as the number of peer-reviewed papers in Scopus x median wage-cost of initial review and re-review).

**Results:**

A total of 354 participants completed at least one question of the survey, and information necessary to calculate the cost of peer-review was available for 308 participants from 33 countries (44% from Canada). The cost of peer review was estimated at $US1,272 per person, per year ($US1,015 for initial review and $US256 for re-review), or US$1.1–1.7 billion for the scientific community per year. The global cost of peer-review was estimated at US$6 billion in 2020 when relying on the Dimensions database and taking into account reviewed-but-rejected manuscripts.

**Conclusions:**

Peer review represents an important financial piece of scientific publishing. Our results may not represent all countries or fields of study, but are consistent with previous estimates and provide additional context from peer reviewers themselves. Researchers and scientists have long provided peer review as a contribution to the scientific community. Recognizing the importance of peer-review, institutions should acknowledge these costs in job descriptions, performance measurement, promotion packages, and funding applications. Journals should develop methods to compensate reviewers for their time and improve transparency while maintaining the integrity of the peer-review process.

**Supplementary Information:**

The online version contains supplementary material available at 10.1186/s41073-023-00128-2.

## Background

Publishing scientific findings is a practice that dates back to 1665 and is the basis for advancing knowledge [1]. The cost of scientific research extends past conducting an experiment, undergoing analysis, or even writing results to be included in an academic paper. There are multitudes of unknown and unaccounted costs associated with scientific research that are not generally discussed, but generally accepted as the cost of “doing business”. In a previous paper, we examined the wage-cost of formatting research papers to be submitted to academic journals [2]. After conducting an online survey, we estimated that each manuscript requires a median formatting time of 14 h, or US$475 to format for publication in a peer-reviewed journal. This represents a loss of 52 h, or a cost of US$1,900 per person-year [2].

After that work was completed, our research team noted that another understudied area was the wage-cost of peer-review for manuscripts submitted to academic journals. Merriam Webster’s dictionary defines peer-review as “a process by which something proposed (as for research or publication) is evaluated by a group of experts in the appropriate field” [3] and since the mid 1900s, peer-review has been considered an important element of the scientific process [4]. When venturing into a career in science, peer-review is generally accepted as an ongoing task, and as an important way to give back to the scientific community [5]. Tendencies on the specifics of peer-review, such as number to complete per year, process for completion, and length of review often vary between people. Differences in review habits may also vary depending on field of study, quality of manuscript, or time available to the reviewer. Some publishers also require more (or less) reviewers before coming to a decision on the publication of a manuscript, and some manuscripts may require many cycles of reviews before being accepted for publication. Many manuscripts will also undergo review at one journal, be rejected, and then go on to be accepted at another journal (after another set of reviews).

In any case, while peer-review is a well-grounded practice, traditionally, peer-review has been an unpaid activity [6] that is generally not captured in performance metrics (e.g., job descriptions, promotion packages, grant reviews) and debates on the validity, reliability, and quality of the peer-review process are common [7]. Some publishers have started to offer compensation in the form of discounts on future publications, or “points” for review credit, but few offer monetary compensation. With peer-review defined as a predominantly voluntary task, and hundreds of research outputs per day requiring peer-review, there is misalignment between the costs gained by publishers and lost by those working and funding science. The fact that academic publishing is a profitable business while institutions pay for access to scientific outputs and reviewers volunteer their time is likely a shocking scenario for those not working in academia (or academic-adjacent positions). As noted in The Guardian, “It is as if the New Yorker or the Economist demanded that journalists write and edit each other’s work for free, and asked the government to foot the bill”, a situation which was once described by Deutsche Bank as “bizarre” [8].

Although we were able to find many opinion pieces on the cost of peer-review [5–8], we were unable to find any study that queried researchers themselves on the time estimated to complete a review. A 2021 analysis by Aczel and colleagues estimated the cost of peer review to be approximately US$2.5 billion in 2020 (US, China and UK-based reviewers combined) [9]. This work is likely the most accurate estimation of the cost of peer-review to date; however, their analysis used publicly available data and their calculations required numerous assumptions that only provide rough estimates of researchers’ time on peer review. In order to address the limitations from Aczel’s study, the present study queries the researchers themselves and aims to get better estimates on the cost of peer review. Specifically, we aimed to provide an updated estimate of the wage-cost of peer-review for scientific publications through a time-use survey of researchers around the world. Secondary objectives included estimating the global cost of peer-review, and assessing if the COVID-19 pandemic had any impacts on peer-review practices.

## Methods

### Study protocol and sample

The Checklist for Reporting Results of Internet E-Surveys (CHERRIES) was used to report elements related to this open survey. A self-report electronic survey (Google Forms) containing 14 questions was sent using a snowball methodology via social media (i.e., Twitter, Facebook), emails, websites and blogs, and word-of-mouth. Social media was the main strategy used to recruit participants. The questions were developed by the authors of this paper and were based on our recent survey that estimated the cost of formatting in scientific publishing [2]. The target population was researchers who acted as peer reviewers for scientific journals in 2020 (convenience sample). This voluntary survey was live between June 28, 2021 and August 2, 2021. No incentives were offered. Due to the snowball sampling methodology, we were unable to determine the response rate and emails of authors were not collected due to the anonymous nature of the survey. Ethics approval was obtained from the Research Ethics Board at the Children’s Hospital of Eastern Ontario Research Institute (file number 20210255 approved on June 9^th^ 2021). Participants provided passive consent by agreeing to take part in the study. The survey took approximately 5 min to complete (estimate) and no personal identifiers were retained. Multiple answers by the same respondent were not possible with this survey. Survey questions can be found in Additional file 1 and raw data can be found in Open Science Framework [10].

The survey was only offered in English; however, there were no geographical limitations on who could answer the questions. There was no minimum or maximum age requirement to participate. There were no exclusions to start the study, or to answer questions related to occupation or salary. However, to proceed further with the survey, only participants who were asked to peer review at least one manuscript submitted in 2020 were able to continue the survey. The survey ended for the other participants.

### Outcomes

The primary outcome measure was the cost of peer review per person, per year (calculated as wage-cost x number of initial reviews and number of re-reviews per year). For the purpose of this work, reviewing was defined as all time related to reviewing manuscripts submitted to peer-reviewed, scientific journals. This included but was not limited to reading the manuscript, making notes, writing the review, proof-reading the review, and completing any forms or questions associated with the submission. Respondents were asked to only count time spent reviewing manuscripts, and not to count time spent reviewing documents for projects internal to their organization, grant proposals, or student theses.

Time spent reviewing was calculated as 1) the time spent on initial review; and 2) the time spent on re-review. Participants were asked to convert their gross annual personal income to US dollars (US$) using an online currency converter [11]. Several participants responded with an income value in their national currency and/or as a specific value. These responses were converted to US$ using R package priceR which accounts for currency values from December 31, 2020 [12]. The mid-point of the income category was used to calculate wage-cost. The wage cost of reviewing was calculated using annual income to estimate wage rates per hour (16 income categories). Hourly rate was based on 1,950 working hours per year. Occupation was used to estimate annual income in cases of refusal responses; however, 99.2% of participants disclosed their annual income.

The global cost of peer-review was estimated as a secondary outcome. Global cost of peer review was calculated first by searching Scopus for all documents published in 2020. Since there was little descriptive information on document type, all documents that were plausibly peer-reviewed were retained for analysis. Number of peer-reviewed documents was multiplied by median wage-cost of an initial review, and re-review. For participants who did not provide an income category (selected the “Other” response item), it was estimated as the median income category for the occupation group with which they identified (*n* = 3). We also examined types of compensation received and changes in review practices due to COVID-19 in exploratory analyses.

In order to better compare with Aczel and colleagues’ paper [9], we conducted another analysis that uses the formula in their paper, based on Publons and the Dimensions database (www.dimensions.ai): (Number of submissions accepted x Average number of reviews accepted) + (Number of submissions rejected x Average number of reviews rejected). According to the Dimensions database, a total of 4,701,988 articles were published in 2020. Aczel and colleagues estimated at 21,800,126 the number of peer reviews for submitted articles in 2020. This number assumes a 55% acceptance rate of reviewed submissions and 45% of submissions that were reviewed but rejected. We then calculated the global cost of peer review by multiplying this number of peer reviews by 6 h for each peer review and by US$46.2 per hour to review (US$90,000 divided by 1,950 h).

### Statistical analysis

To our knowledge, this is the first study that calculates the wage-cost of peer-review for scientific publications by asking the researchers themselves. Our methods were based on a previous study conducted on the wage-cost of formatting [2]. As with our previous analysis, we considered any wage cost associated with peer-review to be significant as it is not normally captured as part of performance reviews or job descriptions, and is typically not included as merit criteria for grant applications or promotions. All variables are summarized as percentage, frequency, and/or median. Interquartile ranges were reported with median values. An audit was performed for outliers, but no results were deemed implausible. Occupation, gender, and country were used for descriptive statistics and subgroup analysis. All analyses were completed using RStudio 1.4.1103 (Boston, MA) and all analyses and results can be found in Open Science Framework [10].

## Results

### Study population

A total of 354 participants completed at least one question of the survey. A small proportion (5.4%, *n* = 19) of respondents reported that they had not been asked to review a manuscript in 2020, and were excluded from the primary analysis. A further 4.5% (*n* = 15) respondents reported that they did not agree to review any manuscripts in 2020, or did not provide an answer for the number of manuscripts reviewed (*n* = 6), or time it took to review (*n* = 6). Information on time for review was available from 308 respondents and this sample was used for the outcome variables. Table 1 shows the summary demographic characteristics of participants surveyed. Participants were from 33 countries around the world, of which 43.9% (*n* = 140) came from Canada. The majority of respondents (73.2%, *n* = 259) reported working as a scientist/researcher (e.g., professor, scientist, researcher, post-doctoral fellow). Approximately half were female and the median age was 40 years. The median annual income category was US$90,000.

**Table 1:** Demographic information of participants (*n* = 354)

**Variable**	**N**	**Proportion (%)**	**Missing**	**Median**	**IQR**
***Demographic variable***					
**Age** (range 25–74)	318	–	36	40	34–47
**Gender**	320	–	34	–	–
Woman	168	52.5	–	–	–
Man	152	47.5	–	–	–
**Country**	319	–	35	–	–
Canada	140	43.9	–	–	–
Australia	45	14.1	–	–	–
United States of America	45	14.1	–	–	–
United Kingdom	24	7.5	–	–	–
Other	65	20.4	–	–	–
**Occupation**	354	–	0	–	–
Scientist/researcher (e.g., professor, scientist, researcher, post-doctoral fellow)	259	73.2	–	–	–
Student (e.g., undergraduate, masters, doctoral)	32	9.0	–	–	–
Clinician/health care provider (e.g., medical doctor, nurse)	19	5.4	–	–	–
Epidemiologist/biostatistician/analyst	14	4.0	–	–	–
Research assistant/research manager	13	3.7	–	–	–
I don’t work in science/academia/research	5	1.4	–	–	–
Other	12	3.4	–	–	–
**Salary**	354	–	0	–	–
< $20,000 per year	14	4.0	–	–	–
$20,000–79,999 per year	147	41.5	–	–	–
$80,000–139,999 per year	152	42.9	–	–	–
$140,000–199,999 per year	20	5.6	–	–	–
≥ $200,000 per year	21	5.9	–	–	–
**Salary (average) (range 10,000–300,000)**	354	–	0	90,000	50 k-130 k
***Outcome variable***					
**Asked to peer-review (during pandemic vs. pre-pandemic)**	333	–	21	–	–
Increased	195	58.6	–	–	–
Stayed the same	109	32.7	–	–	–
Don’t know/can’t remember	19	5.7	–	–	–
Decreased	10	3.0	–	–	–
**Agreed to peer-review (during pandemic vs. pre-pandemic)**	317	–	37	–	–
Stayed the same	134	42.3	–	–	–
Increased	95	30.0	–	–	–
Decreased	75	23.7	–	–	–
Don’t know/can’t remember	13	4.1	–	–	–
**Compensated for peer-review**	320	–	34	–	–
I have never been compensated	280	87.5	–	–	–
Rarely (< 25% of the time)	29	9.1	–	–	–
Sometimes (25–49.99% of the time)	9	2.8	–	–	–
Always (> 75% of the time)	1	0.3	–	–	–
Most of the time (50–75% of the time)	1	0.3	–	–	–

*IQR* interquartile range

### Primary outcome

Wage-cost of peer review is presented in Table 2. Due to positive skewness in the data, median values were used for analysis. We estimated the cost of initial review at US$179 per manuscript, and US$72 for a re-review. The total wage-cost per person, per year was estimated as US$1,272.

**Table 2:** Outcomes related to cost of peer-review for scientific publications (*n* = 308)

**Outcome**	**Per manuscript**	**Per person, per year**
***Number of manuscripts*** (median, (IQR))		
Initial review	-	6 (3–10)
Re-review	-	3 (2–6)
***Time*** (hours; median, (IQR))		
Initial review	4 (3–6)	24 (18–36)
Re-review	2 (1–2)	6 (3–9)
***Cost*** (salary*time)		
Wage-cost of initial review	US$179	US$1015
Wage-cost of re-review	US$72	US$256
**Total wage cost**	–	**US$1272**

Participants were asked about number of manuscripts for the year 2020

*IQR* Interquartile range

### Secondary outcomes and subgroup analysis

Number of manuscripts reviewed by age groups, gender, and occupation are reported elsewhere [10]. Number of manuscripts reviewed was the highest among the 40–49 y age group (median number: 6.5) and among scientists/researchers (median number: 6.5); however, it was the same for men and women (median number: 6 for both). The majority (58.6%) of respondents reported that the number of manuscripts they were asked to review increased from pre-COVID times, whereas 42.3% of respondents reported that the number of manuscripts they agreed to review stayed the same. The large majority of respondents (87.5%, *n* = 280) reported that they never received any compensation for their review; some (9.1%, *n* = 29) reported that they rarely (i.e., < 25% of the time) receive compensation; and a small proportion (2.8%, *n* = 9) reported that they sometimes (i.e., 25–49.99% of the time) receive compensation. Compensation was most often a discount on publishing fees for a specific journal, or access to journal libraries for specified time periods.

Globally, Scopus results show 3,545,399 documents for the year 2020 [13]. Taking a very conservative approach, and only including documents indexed as “article” (*n* = 2,514,881) and assuming two reviewers per paper and one re-review, the cost of peer-review is US$1,081,398,830 per year globally. If we take a somewhat less conservative approach and include documents tagged as articles (*n* = 2,514,881), reviews (*n* = 222,112), book chapters (*n* = 65,684), short surveys (*n* = 9,489), books (*n* = 4,090) and data papers (*n* = 2,509), and assume three reviewers and one re-review, the global cost of peer-review increases to US$1,716,627,885. It should be noted that our survey asked specifically about “manuscripts”, but this less conservative approach includes all documents that plausibly went through the peer-review process.

According to the Aczel and colleagues’ formula [9], they estimated the total number of peer reviews per year at 21,800,126. By assuming 6 h for each peer review and US$46.2 per hour to review (US$90,000 divided by 1,950 h), the global cost of peer-review is estimated at US$6,042,994,927 in 2020. Of note, this calculation relies on the Dimensions database (~ 87,000 scholarly journals vs. ~ 20,000 for Scopus) and takes into account reviewed-but-rejected manuscripts (45% of all reviewed submissions according to Publons), which explains the much larger global estimate.

## Discussion

To our knowledge, this is the first study that directly surveys reviewers to estimate the time and wage costs associated with peer-review in scientific publishing. Our results suggest that peer-review represents an important financial piece of scientific publishing, taking approximately 4 h for an initial review and 2 h for a re-review. Respondents reported they reviewed approximately 6 papers per year and re-reviewed 3 papers per year. Therefore, based on data reported to us, we estimate the cost of peer-review to be approximately US$1,272 per person, per year, and represents a cost of approximately US$1.1–1.7 billon dollars to the scientific community per year (conservative approach), or US$6 billion in 2020 when using the Dimensions database and including reviewed-but-rejected manuscripts.

Our results of a wage-cost of US$179 per review is slightly lower than previous estimates of US$250–450 per review [14–16]; however, our results seem to be the first to survey peer-reviewers directly. Our estimates are also similar to those from Aczel et al. [9], who calculated an hourly review cost of US$69.25/hour for U.S. based researchers, US$57.21/hour for U.K. based researchers, and US$33.26 for China-based researchers, which equates to a peer-review cost of US$277, US$228.84, and US$33.04, respectively. The difference in cost was due to differences in median salaries across countries. We took the salary for each individual respondent whereas Aczel took the average salary between a senior researcher and a junior researcher. While lower than previous estimates, a wage-cost of US$179 should be considered a significant cost, given its voluntary nature, and especially as many have estimated that the publishing business generates billions of dollars per year with profit margins at 20–30% for the industry [8, 17]. We hope that this paper will raise awareness on this subject and stimulate discussion and future studies.

People generally complete numerous reviews (and re-reviews) per year, and each manuscript requires multiple reviews (and re-reviews), many times at numerous journals, before being published. So the problem is not just the cost of a single review, but the overall cost to the scientific community. According to the 2018 Global State of Peer Review by Publons [18], article publication volumes have grown by 2.6% per year while submissions have grown by 6.1% per year since 2013. Based on this report, the median time spent writing each manuscript review in 2016 was 5 h, in line with what we reported in the present study.

Our results suggest an average of six initial reviews, and three re-reviews per year. This results in a significant personal cost per year, and an astonishing cost of global peer-review annually. After consultation with a variety of sources (e.g., institutional librarians, content experts), we were unable to find a very accurate number of published peer-reviewed papers in a single year. Web of Science (subscription based), 1science’s 1findr, Digital Science’s Dimensions, and Informa’s wizdom.ai provide estimates of annual scientific publications [19], but no one source seemed to contain all possible information, and it was not feasible to systematically scan and cross-reference all databases to determine a precise publication count. We were also unable to find any information on typical review practices (i.e., number of reviews, re-reviews, and peer-review time estimates) from any major journal. Moving forward, journals should make this information publicly available so we can more accurately quantify the cost of peer-review.

It is clear that changes need to be made to make peer-review more equitable, more feasible, and more stable. We are not advocating for any one solution, but would like to provide some recommendations for individuals, institutions, funding agencies, and journals. These suggestions are aimed at improving the review process for individuals, and ultimately with improving overall quality and consistency of peer-review and associated academic outputs.

At the individual level, some have suggested a solution is to pay peer-reviewers for their time; however, 87.5% (*n* = 320) of our survey respondents reported that they have never received any type of compensation. A modest compensation (e.g., US$50 per review) may entice reviewers to contribute more often [20]. For example, an hourly pay for grant evaluations has been proposed [21] and a recent study on double blind peer review in the finance field stated that it is standard practice for the journal to pay reviewers US$50 for review, regardless of hours needed to do the work or country of origin [22]. This may also be a way to “level the playing field” between the multi-billion-dollar publishing industry and those completing the reviews [8]. It may also provide an incentive to younger researchers, and/or those with lower incomes, to participate in the peer-review process. However, paying reviewers may be complex and can come with its own limitations [23]. For example, should compensation be the same regardless of the length or complexity of the manuscript, or should it be on a sliding scale [23]? Compensation could also theoretically lead to poor quality reviews (e.g., too short, unhelpful, or under-critical), or unethical behaviour (e.g., showing preference to some reviewers) and therefore may jeopardize the essence of the peer-review process [23]. However, these concerns could be addressed by putting safeguards in place; many journals already provide reviewers with a review template and guidelines, and could stipulate that only reviews meeting those guidelines will receive compensation. Reviewers may also be required to include a more detailed disclosure statement to identify any perceived or real conflicts of interest (e.g., in the event that an editor is showing preference for one reviewer). It is also possible that additional costs associated with peer-review would be passed on to journal subscribers (e.g., libraries or institutions), which may increase disparities in the scientific process [23].

Others have suggested to limit the number of articles a person reviews in a year. While this seems reasonable, the number of publications submitted for peer-review, and therefore the number of peer reviewers needed, is growing exponentially. In 2014, there was an annual increase of 8–9% in the number of publications, translating to a doubling of scientific output every nine years [24]. More recent reports suggest global scientific output in 2019 was 21% higher than that in 2015 [25]; so, if reviewers simply declined to act as peer-reviewers, the scientific process may be quickly immobilized. Although a peer-review strike may provide a compelling reason for academic publishers to re-consider current approaches, it is likely not the best situation for the advancement of science. A more conservative approach may be to rely more heavily on alternate publication structures (e.g., online repositories), thereby circumventing for-profit models. However, this may also lead to the degradation of the current peer-review structure.

Institutions also play an important role in the peer-review process by setting realistic expectations for the number of peer-reviews done per year, the way in which they are handled, and the acknowledgement they receive (e.g., in terms of performance reviews or promotional packages). Job descriptions should clearly explain expectations related to peer-review and even provide recommendations appropriate for various employment situations (e.g., a new investigator may be required to review less than someone with an established research lab and/or staff). Funding agencies may add this item to grant applications through investigator biographies and acknowledge those who are contributing to their field. Funding agencies may also imbed peer-review training into their fellowship programs, to help develop best practices among early career scientists. Institutions and funding agencies could also set internal policies with clear recommendations for compensation for peer-review. For example, just as some funding agencies require that any resulting manuscripts be published in open access journals, they could also require that results be submitted to journals that enact best practices in compensation for peer-review.

It is also important to note that we did not ask reviewers if they performed their reviews during working hours. Although peer review can be seen as a pro-bono/volunteer work for researchers as their contribution to the society and science, it is likely that the majority of researchers conduct peer reviews as part of their job. Considering that most reviewers are being paid for this work by their employees and therefore is part of their job, it would not be unreasonable to have this in the contract when universities hire professors/researchers. Based on our study, time to peer review is estimated at 30 h per year per researcher, the equivalent to almost 1 week of paid work.

When estimating the cost of peer review, it is important to consider two interests whose logic is completely different. The first is to analyse the loss of earnings for researchers (or the savings made by publishers) if this activity were to be paid for. The second is to estimate the price of peer review, if this activity becomes remunerated in the future. The calculations carried out in this study are the product of the crossing between the time spent peer reviewing and the salary of the reviewers. However, if we were in a logic of remunerating reviewers in the future, only the time spent will be used in the calculation. The reason being that the quality of a reviewer’s report does not necessarily depend on their salary. Future analyses should also cross-reference the time spent peer reviewing and the number of words (or pages) written, because not all reviewers have the same productivity/efficiency. The time spent peer reviewing is not necessarily a good indicator of the quality of the reports produced.

Journals and publishers need to think creatively to compensate reviewers, provide realistic expectations for reviews, facilitate the review process, ensure quality reviews and improve transparency. It is not uncommon for reviewers to be asked to review just before a major holiday and then a reminder 1 week later. It may also mean that journals keep track of how many times they have reached out to a reviewer and limit their invitations to only when the manuscript is a close fit to the reviewer’s area of expertise. Publishing houses may offer mentorship and training sessions to facilitate the review process, especially for those who have limited experience with completing a review. Napoliatani et al. [4] have written suggestions for both reviewers and journals on this topic. Journals should also provide more transparency in the peer-review process, including costs associated with publication. It is largely unclear how many reviewers each journal requires, the average length of review, how many people they have asked to review per year, and/or how many people have agreed to review per year. It is also largely unclear how much it costs for a journal to publish a paper (e.g., editors, administrative staff, distribution and publishing), with most journals unwilling to make this information publicly available [20]. This information is crucial in understanding the cost of peer-review and an important part of the scientific process.

## Limitations

As with any study, this work has several strengths and limitations. We were able to recruit a relatively large sample across 33 countries; however, we did not collect any information on field of study, leaving us to wonder if peer-review habits vary across disciplines. Time devoted to peer review depends on the technicality of the papers and their discipline; this should be assessed in future studies. Further, 44% of our population came from Canada, which further limits the generalizability of our results. Future work should focus on establishing a sample that is more representative of the international publishing landscape. Based on the Publons Global State of Peer Review survey [18], reviews are supplied in this order of countries: USA (23%), China (7%), UK (7%), Japan (4%), Germany (3%) and Canada (2.5%). Our survey is thus skewed with an overrepresentation of scientists from Canada. Participants also came from various levels of employment and had a range of salaries, suggesting a cross-section of the scientific community. We did not ask about review experience, and it is possible that those who received some sort of mentorship and/or training were able to complete reviews more efficiently. This work was based on a short self-report sample and our data collection was based on snowball sampling of a convenience sample, thereby likely biasing our results. Specifically, it is possible that some respondents over-estimated the number of manuscripts they reviewed and the time it took them to review; however, we tried to address this through our analytical plan and the results presented herein seem to be a reasonable depiction of reality and are relatively consistent with previous estimates. Future work may focus on validating this type of questionnaire. We also required participants to have agreed to review at least one manuscript during 2020, which may not represent an “average” year due to the COVID-19 pandemic, although 42.3% of respondents said they agreed to review the same number of manuscripts as pre-pandemic. The survey was only available for a short time frame and it is possible that with a longer study period, or longitudinal analysis, we would uncover different results. Future work may address these shortcomings. Finally, in researching for this paper, we came across very few academic papers addressing the topic of cost in peer-review. We were able to find a significant number of blog posts, online commentaries, and even some editorials in academic journals, but, somewhat ironically, the debate on cost of peer-review has been done largely outside the peer-review system.

In summary, we estimate the cost of peer-review for scientific publication at US$1,272 per person, per year. Globally, this may account for between US$1.1–1.7 billion dollars annually (conservative estimate) or US$6 billion in 2020 when using the Dimensions database and including reviewed-but-rejected manuscripts. Our estimations only represent rough orders of magnitude given the snowball sample used and the many uncertainties in the data. However, we hope that this quantification will bring more attention to the costs associated with peer-review and that institutions, funding agencies, and publishers will support scientists to find more amenable solutions in the future. Recognizing the importance of peer-review, institutions should acknowledge these costs in job descriptions and performance measurement, and advocate for a more equitable partnership with academic publishers. Research agencies should account for this essential service in grant applications. Journals and publishers should develop methods for remuneration that both compensate reviewers for their time and maintain the integrity of the peer-review process.

## Supplementary Information

**Additional file 1.** Survey questions.

## Data Availability

The public dataset supporting the conclusions of this article is available here: https://osf.io/bfhgd/?view_only=41a4f3566b7c44d49a90842521b5c976.
