# Supplementary material for: Scientific sinkhole: estimating the cost of peer review based on survey data with snowball sampling
Source: Res Integr Peer Rev. 2023 Apr 24;8:3. doi: 10.1186/s41073-023-00128-2 (PMC10122980; doi:10.1186/s41073-023-00128-2)
Supplement: Supplementary file 1 — Additional file 1. Survey questions. [file 41073_2023_128_MOESM1_ESM.docx]

**Additional file 1.** Survey questions.

The following questions relate to your occupation. We require your estimated income to calculate the hourly cost attributable to reviewing publications for academic journals. Although we understand this is a personal question, your answer will be kept anonymous, and used only to calculate costs related to reviewing manuscripts.

1. What best describes your occupation in 2020 [UNIQUE ANSWER]
   1. Clinician/health care provider (e.g., medical doctor, nurse)
   2. Scientist/researcher (e.g., professor, scientist, post-doctoral fellow)
   3. Research assistant/research manager
   4. Epidemiologist/biostatistician/analyst
   5. Student (e.g., undergraduate, masters, doctoral)
   6. Other [OPEN FIELD]
   7. I don’t work in science/academia/research [END SURVEY]
2. What was your gross personal income in 2020 (please estimate in U.S. dollars; if unsure, please enter the currency in which you are paid). You can access a currency converter here: https://www.xe.com/currencyconverter/
   1. <$20,000 per year
   2. $20,000-39,999 per year
   3. $40,000-59,999 per year
   4. $60,000-79,999 per year
   5. $80,000-99,999 per year
   6. $100,000-119,999 per year
   7. $120,000-139,999 per year
   8. $140,000-159,999 per year
   9. $160,000-179,999 per year
   10. $180,000-199,999 per year
   11. $200,000-219,999 per year
   12. $220,000-239,999 per year
   13. $240,000-259,999 per year
   14. $260,000-279,999 per year
   15. $280,000-299,999 per year
   16. ≥$300,000 per year
   17. ____ Other (specify currency)

The following questions refer to manuscripts that you were asked to peer-review **in 2020**. Due to the COVID-19 pandemic, we realize that the number of manuscripts you have been asked to review, or agreed to review, may have varied due to a variety of factors.

Please think of only times you acted as an external peer-reviewer and assessed a manuscript that was submitted to an academic journal (e.g., Nature, PLOS ONE). When we ask about reviewing, we are referring to all time related to reviewing including but not limited to reading the manuscript, making notes, writing the review, proof-reading your review, and completing any forms or questions associated with the submission.

DO NOT count time spent reviewing documents for projects internal to your organization, grant proposals, student thesis, etc.

1. On average, how often are you **asked** to review a manuscript for a peer reviewed, academic journal? DO NOT count requests from suspected predatory journals.
   1. ____ time(s) per year
   2. Never [END SURVEY]
2. Has the number of manuscripts you are **asked** to review increased, decreased, or stayed the same during the COVID-19 pandemic (i.e., since March 2020) compared to before?
   1. Increased
   2. Decreased
   3. Stayed the same
   4. Don’t know/can’t remember
3. Thinking of all manuscripts you were asked to review in 2020, how many did you **agree** to review?
   1. _____
   2. 0 [END SURVEY]
4. Has the number of manuscripts you have **agreed** to review increased, decreased, or stayed the same during the COVID-19 pandemic (i.e., since March 2020) compared to before?
   1. Increased
   2. Decreased
   3. Stayed the same
   4. Don’t know/can’t remember
5. On average, how much time does it take you to review a single manuscript (e.g., reading, taking notes, writing the review, proof-reading your submission, completing forms and questions)?

DO NOT count time spent re-reviewing a manuscript (i.e., reviewing the same manuscript for a second time pre-publication).

- 1. _____ [hour(s)]

1. In 2020, how many manuscripts did you agree to **re-review**?

Note: re-reviewing is when you are asked to review a manuscript for a second time after the authors have made edits and/or responded to your comments from a previous review, pre-publication.

- 1. _____

1. On average, how much time does it take you to **re-review** a single manuscript (e.g., reading, taking notes, writing your comments, proof-reading your submission, completing forms and questions)?
   1. _____ [hour(s)]
2. Are you ever compensated for reviewing manuscripts? Compensation can come in many forms but please DO NOT count discounts to publish in a specific journal.
   1. Always (>75% of the time)
   2. Most of the time (50-75% of the time)
   3. Sometimes (25-49.99% of the time)
   4. Rarely (<25% of the time)
   5. I have never been compensated [SKIP QUESTION 11]
3. What type of compensation have you received?
   1. ­­­Open text box
   2. Prefer not to answer

Finally, some questions on general demographic information.

1. What is your age?
   1. _____ years
2. What is your gender?
   1. Woman
   2. Man
   3. Other
   4. Prefer not to answer
3. What country do you work in the majority of the time?
   1. [dropdown list of countries]
